# Caregiver burden in lung transplantation: A review

**DOI:** 10.1016/j.jhlto.2025.100239

**Published:** 2025-03-04

**Authors:** Keerthana Sankar, Anne Johnson, Kathryn McRae, Reinaldo Rampolla, Nicholas A. Kolaitis

**Affiliations:** a Division of Pulmonary and Critical Care, Cedars-Sinai Medical Center, Los Angeles, California; b Lung Transplant Program, University of California, San Francisco, California; c Division of Pulmonary, Critical Care, Allergy and Sleep Medicine, Department of Medicine, University of California, San Francisco, California

**Keywords:** caregiver, caretaker, lung transplant, pre- and post-transplant, solid organ transplant

## Abstract

Caregivers play a pivotal role in supporting patients undergoing lung transplant and often experience significant psychological, physical, and financial burdens. The current literature suggests that caregivers face heightened depression and anxiety in both the pre- and post-transplant periods. Caregivers of lung transplant patients may face unique challenges compared to other caregiver populations due to fears regarding pre- and post-transplant survival, lifestyle changes associated with post-transplant complications, and financial burden due to job loss and relocation. Caregiver well-being is linked to patient outcomes, with increased burden associated with higher post-transplant mortality. This review summarizes the symptoms associated with caregiver burden, assessment tools to evaluate caregiver strain, patient outcomes and social determinants of health as it relates to caregiver-patient relationships, and interventions to mitigate caregiver burden in the lung transplant population.

## Background

In solid organ transplantation, lung transplantation is the organ subgroup with highest risk of mortality.^1^ Patients awaiting lung transplant experience physical and psychological symptoms, and after transplantation, require frequent outpatient follow-up, complex medication management, and face potential post-transplant complications.^2^ Caregivers play an important role before and after lung transplantation. The International Society for Heart and Lung Transplantation 2021 consensus statement endorses that adequate social support and caregiving are critical for successful outcomes and that lack of support may increase post-transplant mortality.^3^ Based on recommendations from the organ transplant caregiver initiative, a patient usually cannot be listed for transplant without designating at least one full-time caregiver.^4^ Given the emotional, financial, and psychosocial stressors associated with lung transplant, quality of life (QOL) in caregivers is negatively impacted, and caregiver stress is associated with worse post-transplant outcomes.^5^, ^6^, ^7^, ^8^, ^9^, ^10^

In this review focused on caregiver burden in lung transplantation, we delineate symptoms of caregiver burden, evaluate assessment tools of caregiver strain, identify the impact of caregiver strain on patient outcomes, describe the intersection between caregiver strain with social determinants of health, and report interventions to mitigate caregiver burden.

## Methods

This narrative review was written based on a comprehensive literature search identifying studies related to caregiver burden using PubMed, Ovid, and Embase. These databases were searched with the following queries: “lung transplant AND caregiver,” “solid organ transplant AND caregiver,” “psychological distress AND lung transplant,” “caregiver interventions and lung transplant,” and “caregiver outcomes AND lung transplant.”

**Figure 1:**
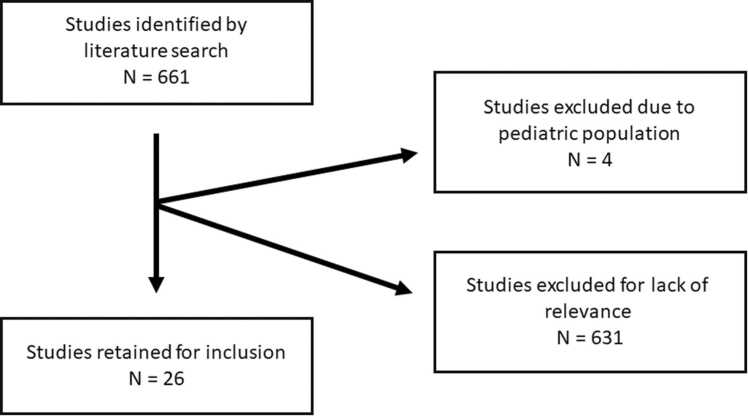
Flowchart of literature search.

Additional articles were identified by references cited in the publications found. Titles and abstracts were read in full and screened by 2 reviewers (K.S. and N.A.K.). Relevant articles were read in full by one reviewer (K.S.) and validated for relevance by a second reviewer (N.A.K.). Prespecified exclusion criteria were studies written in a language other than English, studies conducted in pediatric patients, and studies that were not relevant to caregiver burden. We identified 661 articles across all search modalities (PubMed = 228, Ovid = 68, and Embase = 365) of which 26 directly assessed caregiver strain in lung transplantation (Figure 1). Relevant studies in other solid organ transplantation were also included in the absence of evidence in the lung transplantation population. Studies regarding interventions in the caregiver population and caregiver outcomes in the nontransplant population, which were also found in the search, were also included to supplement emerging literature in the transplant population.

### Lung transplantation and mental health

Patients undergoing lung transplantation face mental health challenges in the pre- and post-transplant periods. Before transplantation, patients may experience worsening symptoms and functional impairments that affect QOL.^2^, ^11^ Mood disorders have a varied prevalence in the pre- and post-lung transplant populations. In patients awaiting lung transplant, a cross-sectional study of 100 patients with end-stage lung disease demonstrated that 25% met diagnostic criteria for at least one mood or anxiety disorder.^11^ Multiple studies demonstrate a correlation between post-transplant mood disorders and increased incidence of declining lung function, chronic rejection, and mortality.^12^, ^13^, ^14^, ^15^

Caregivers are instrumental in supporting patients in the pre- and post-transplant period and can be protective against psychological symptoms and post-transplant complications. A study conducted on 76 lung, liver, and bone-marrow transplant candidates demonstrated that poor social support is an important component of psychosocial vulnerability and a predictor of poor general life satisfaction after transplant.^16^ Another qualitative review in solid organ transplantation demonstrated that psychosocial status generally improves with transplant and that good social support has an additional protective effect on mental health.^17^

### Characteristics of caregiver burden in the pretransplant period

Our literature search identified 26 studies evaluating caregivers in the lung transplant population, 19 of which pertained to caregiver burden in the lung transplant population (Table 1). Of those pertaining to caregiver burden, 6 studies were conducted in the pretransplant population, 7 studies in the post-transplant population, and 6 studies spanning before and after transplantation. All articles directly assessing caregiver burden which reported gender contained a higher proportion of female caregivers^5^, ^6^, ^7^, ^8^, ^18^, ^19^, ^20^, ^21^, ^22^, ^23^, ^24^, ^25^, ^26^, ^27^, ^28^, ^29^ with the exception of one study which reported an equal male-to-female ratio.^30^ Mean caregiver age above 50 was most commonly reported.^5^, ^7^, ^8^, ^18^, ^19^, ^20^, ^21^, ^22^, ^23^, ^24^, ^25^, ^26^, ^27^, ^30^

**Table 1:** Primary Studies Describing Caregiver Burden in Lung Transplantation

Year	Author	Title	Cohort size	Transplant population	Pre/post transplant	Mean caregiver age	Caregiver male/female ratio	Location
1992	Saxe-Braithwaite et al	Life on hold: the experience of the support person involved in a lung transplant program	5	Lung	Pre and post	unknown	40/60	United States
2001	Meltzer et al	Psychological distress in caregivers of liver and lung transplant candidates	52	Lung/liver	Pre	54.79	29.2/70.8	United States
2005	Claar et al	Emotional distress and quality of life in caregivers of patients awaiting lung transplant	82	Lung	Pre	47.6	20.7/79.2	United States
2005	Myaskovsky et al	Quality of life and coping strategies among lung transplant candidates and their family caregivers	114	Lung	Pre	42-57^a^	60.5/39.5	United States
2006	Rodrigue et al	Caregivers of lung transplant candidates: do they benefit when the patient is receiving psychological services?	28	Lung	Post	Unknown	Unknown	United States
2006	Goetzmann et al	Psychosocial vulnerability predicts psychosocial outcome after an organ transplant: Results of a prospective study with lung, liver, and bone-marrow patients	76	Lung/liver/bone marrow	Pre	N/A	N/A	Switzerland
2007	Rodrigue et al	Waiting for lung transplantation: quality of life, mood, caregiving strain and benefit, and social intimacy of spouses	73	Lung	Pre	48.3	46.6/53.4	United States
2008	Gries et al	Caregivers of lung transplant recipients exhibit high risk for psychological symptoms	31	Lung	Post	58^b^	20/80	United States
2009	Lefaiver et al	Quality of life in caregivers providing care for lung transplant candidates	29	Lung	Pre	50.43	34/66	United States
2010	Song et al	Exploring the meaning of chronic rejection after lung transplantation and its impact on clinical management and caregiving	10	Lung	Post	Unknown	70/30	United States
2011	Goetzinger et al	Stress and coping in caregivers of patients awaiting solid organ transplantation	621	Lung/liver/heart/kidney	Pre	51.7	73/27	United States
2012	Xu et al	Daily burdens of recipients and family caregivers after lung transplant	21	Lung	Post	52.3	48/52	United States
2012	Myaskovsky et al	Predictors and outcomes of health-related quality of life in caregivers of cardiothoracic transplant recipients	242	Lung/heart	Post	Unknown	34.3/65.7	United States
2013	DeVito et al	Quality of recipient-caregiver relationship and psychological distress are correlates of self-care agency after lung transplantation	112	Lung	Post	Unknown	Unknown	United States
2013	Dew et al	Onset and risk factors for anxiety and depression during the first two years after lung transplantation	304	Lung/heart	Post	N/A	N/A	United States
2014	Haines et al	Reducing stress and anxiety in caregivers of lung transplant patients: benefits of mindfulness meditation	30	Lung	Post	55.6	23/77	United States
2014	Ivarsson et al	Relative's experiences before and after a heart or lung transplantation	15	Lung/heart	Post	51	50/50	Sweden
2015	Mollberg et al	Impact of primary caregivers on long-term outcomes after lung transplantation	452	Lung	Post	Unknown	Unknown	United States
2016	Haines et al	Tales of roller coaster rides and resilience: lung transplant caregivers in their own words	60	Lung	Post	Unknown	Unknown	United States
2016	Li et al	Lung transplant patients and carers report unmet palliative care needs	87	Lung	Pre and post	60	48/52	Australia
2017	Agren et al	Psychosocial aspects before and up to 2 years after heart or lung transplantation: experience of patients and their next of kin	40	Lung/heart	Pre and post	51	31.5/68.4	Sweden
2020	Yagelniski et al	A qualitative study to explore the needs of lung transplant caregivers	12	Lung	Pre and post	64.6	8.3/91.7	Canada
2021	Pawlow et al	The supportive care needs of primary caregivers of lung transplant candidates	78	Lung	Pre	58	35.1/65.6	United States
2021	Glaze et al	The lived experiences of caregivers of lung transplant recipients	20	Lung	Pre and post	51.6	45/55	United States
2021	Giordano et al	Assessing how a transplant hospitality house for patients and families can promote wellbeing	15	Lung/liver/kidney	Pre and post	Unknown	33.3/66.7	United States
2023	Yildizeli et al	Burden, depression and fatigue in caregivers of lung transplantation candidates	39	Lung	Pre	41.8	20.5/79.5	Turkey

a Interquartile range.

b Median.

In the pretransplant caregiver population, clinical depression and anxiety were noted. A cross-sectional study of 82 caregivers who completed psychosocial questionnaires before patients’ pretransplant psychosocial evaluation showed that 12 (14.6%) and 2 (2.4%) caregivers had clinically significant levels of depression and anxiety, respectively.^19^ In another study conducted on 621 caregivers of patients undergoing any solid organ transplantation, 17% exhibited symptoms of depression and 13% reported anxiety. No differences in symptom burden were found across transplant type.^31^ Although these studies did not contain direct comparison groups, the prevalence of depression and anxiety was higher than estimates of the population prevalence (9.2% depression and 6.7% anxiety).^32^, ^33^ The gap is even wider when compared to the population prevalence of depression above the age of 50 (<6%).^33^

Many aspects of the pretransplant period contribute to heightened levels of mood disorders in caregivers. The multidisciplinary evaluation can be extremely stressful as it is the patient’s and caregiver’s only hope for extended survival. The patient’s health may also continue to decline at this time resulting in more caregiver responsibility for day-to-day tasks. Patients and caregivers may also struggle with the idea of receiving an organ from another person and experience guilt that someone must die for the patient to live.^21^, ^29^ Mood disorders in caregivers may also be under-reported in the lung transplant literature as caregivers may fear jeopardizing transplant candidacy.

Caregivers in the pretransplant population also reported fears of financial instability. One study noted that caregivers of lung transplant patients wanted more support for financial, legal, or work issues when compared to other groups of seriously ill patients.^18^ Another study noted that impact on finances had the greatest effect on caregivers’ QOL.^5^ Many caregivers relocate during the first year after transplantation. In a study of 10 patients receiving heart transplants and 8 caregivers, the median time away from home was 14 months, 63% of caregivers faced financial strain resulting in debt or bankruptcy, and 87% of caregivers felt that psychological support during time of relocation was lacking.^34^ Oftentimes, caregivers and patients must relocate before transplantation to have timely access to the hospital when a suitable donor is found and to prepare for post-transplant care which includes rehabilitation and frequent follow-up visits. This adds to the duration of time away from home and increases both financial and emotional stress as caregivers are unable to work and are separated from local support systems.^31^ Relocation rates are likely higher in regions and countries where there are fewer transplant centers and may result in increased financial burden. Studies describing caregiver burden associated with relocation in the lung transplant population are needed to identify ways in which transplant programs can provide support during this time.

The relationship between patients and caregivers plays an important role in caregiver QOL. In a study of 73 caregiver spouses of patients awaiting lung transplant, 56.2% reported clinically significant levels of caregiver strain in the following areas: physical strain, inconvenience, feeling confined, and feeling upset by how much the patient had changed. Compared to a healthy sample of 1,982 adults, QOL scores were significantly lower, indicating that spouses may uniquely experience significant burden.^7^ Coping strategies of patients and caregivers are pertinent to QOL and management of stress. A cross-sectional study of 114 adult patients awaiting lung transplant and their caregivers revealed that patients who used emotionally-oriented coping (greater support seeking, avoidant behavior, and self-blame) had poorer QOL, as did their caregivers.^35^

Lastly, patients’ physical health correlates with caregiver strain in the pretransplant period. A cross-sectional study of 78 caregivers showed that the Lung Allocation Score, which correlates with severity of illness, was the most highly associated factor for increased caregivers’ supportive needs.^18^

### Characteristics of caregiver burden in the post-transplant period

After lung transplantation, caregivers face challenges associated with postoperative complications, hospital readmissions, medication management, frequent clinic visits, and fear of chronic rejection or death. In the immediate post-transplant period, patients are monitored in the intensive care unit (ICU) and require mechanical ventilatory support. Multiple hospital readmissions are also common among lung transplant patients and may require additional ICU stays. Studies have demonstrated that caregivers of patients admitted to the ICU experience high levels of both physical and psychological symptoms.^36^, ^37^ Among 50 caregivers of critically ill patients who required mechanical ventilation for more than 4 days, 94% reported health-risk behaviors, including inadequate rest, skipping meals, lack of exercise, and difficulty taking medications.^36^ Though these studies were not conducted in post-transplant ICU patients, uncertainty regarding the post-transplant course is a known source of caregiver distress.^29^

After returning home, caregivers continue to face emotional and physical strain. A study examining the daily emotions and moods of 21 lung transplant recipients and their caregivers demonstrated that caregivers reported positive emotions more often than patients though reported overall lower daily mood. This may be attributed to caregivers finding a sense of purpose despite the toll on overall mental health. Additionally, 15% reported that their health had worsened since taking on caregiving responsibility.^8^ Several studies demonstrate the correlation between caregiving and declining physical health. In a study of caregivers for 242 cardiothoracic transplant recipients, caregiver QOL was high during the first year after transplant in emotional and social functioning, but physical functioning and bodily pain worsened over time.^6^ In the non–lung transplant population, caregiving was independently associated with increased risk of coronary artery disease and hypertension.^38^, ^39^, ^40^ Studies assessing physical manifestations of caregiver strain are needed in the pre- and post-lung transplant population.

Importantly, chronic rejection and death are inevitable in the post-transplant period and can cause a re-emergence of increased caregiver burden in the later years after transplant. A study evaluating caregivers’ perspective of chronic rejection in lung transplant recipients demonstrated that most caregivers equated chronic rejection with death which shifted their mindset to the pretransplant period. Caregivers also reported that their responsibilities increased and became more complex after a diagnosis of chronic rejection due to patients developing more symptoms, requiring oxygen, and becoming less independent.^41^

The post-transplant period is complicated and unpredictable in lung transplant patients which can cause dramatic shifts in caregiver responsibility. As lung transplant recipients have the highest rate of rejection and shortest survival of all organ transplants, additional studies are necessary to determine the unique caregiver burden of this population at various time points after transplant.^42^

### Assessment methods of caregiver strain

Multiple assessment methods can evaluate caregiver burden. Of the 19 studies directly assessing caregiver burden in the lung transplant population, more than 15 instruments were used (Table 2). In a systematic review assessing the reliability and validity of caregiver-specific instruments across various populations, 74 screening tools were identified within 112 studies.^43^ While a number of tools, such as the Zarit-Caregiver Burden Scale, Caregiver Reaction Assessment, and Caregiver Strain Index, focus on negative effects of caregiving, others such as the Cares of Older People in Europe (COPE) Index assess both the positive and negative effects of caregiving.^43^, ^44^ In studies pertaining to caregivers in lung transplantation, caregiver-specific instruments were used in conjunction with non–caregiver-specific instruments.^5^, ^7^, ^19^, ^20^, ^22^, ^45^, ^46^, ^47^, ^48^ Some studies used semistructured interviews to evaluate caregiver burden.^24^, ^27^, ^28^, ^30^ While these interviews capture the complexity of caregiver burden, they lack validity due to diverse responses among study participants which can limit interpretation.^49^

**Table 2:** Caregiver Burden Assessment Tools in the Lung Transplant Population

Author	Title	Assessment instruments
Saxe-Braithwaite et al	Life on hold: the experience of the support person involved in a lung transplant program	Unstructured, informal interviews
Meltzer et al	Psychological distress in caregivers of liver and lung transplant candidates	Short Form (SF)-36, Psychosocial Adjustment To Illness Scale-Self Report, Caregiver Strain Index
Claar et al	Emotional distress and quality of life in caregivers of patients awaiting lung transplant	Beck Depression Inventory-II, State-Trait Anxiety Inventory (STAI), Medical Coping Modes Questionnaire, Scale for Caregiver Burden, SF-36
Rodrigue et al	Caregivers of lung transplant candidates: do they benefit when the patient is receiving psychological services?	QOL inventory, Profile of Mood States-short form (POMS-SF), Miller Social Intimacy Scale
Rodrigue et al	Waiting for lung transplantation: quality of life, mood, caregiving strain and benefit, and social intimacy of spouses	QOL Inventory, SF-36, POMS, Caregiver Strain Index, Caregiver Benefit Index, Miller Social Intimacy Scale
Gries et al	Caregivers of lung transplant recipients exhibit high risk for psychological symptoms	Posttraumatic Stress Disorder Checklist, Patient-Health Questionnaire-9, Mental Health Questionnaire
Lefaiver et al	Quality of life in caregivers providing care for lung transplant candidates	QOL Index, SF-12, POMS-SF, Caregiver Reaction Assessment
Xu et al	Daily burdens of recipients and family caregivers after lung transplant	Day Reconstruction Method
Myaskovsky et al	Predictors and outcomes of health-related quality of life in caregivers of cardiothoracic transplant recipients	SF-36, 28-item brief Coping Orientation to Problems Experienced scale
Haines et al	Reducing stress and anxiety in caregivers of lung transplant patients: benefits of mindfulness meditation	Perceived Stress Scale (PSS), STAI
Ivarsson et al	Relative's experiences before and after a heart or lung transplantation	Semistructured interviews
Haines et al	Tales of roller coaster rides and resilience: lung transplant caregivers in their own words	PSS, STAI
Li et al	Lung transplant patients and carers report unmet palliative care needs	SF-36, Edmonton Symptom Assessment System, Carer Support Needs Assessment Tool
Agren et al	Psychosocial aspects before and up to 2 years after heart or lung transplantation: experience of patients and their next of kin	EuroQol 5-dimensional questionnaire, Hospital Anxiety and Depression Scale, Impact of Event Scale, Mastery Scale, Caregiver Burden Scale
Yagelniski et al	A qualitative study to explore the needs of lung transplant caregivers	Semistructured interviews
Pawlow et al	The supportive care needs of primary caregivers of lung transplant candidates	Carer Support Needs Assessment Tool
Glaze et al	The lived experiences of caregivers of lung transplant recipients	Semistructured interviews
Giordano et al	Assessing how a transplant hospitality house for patients and families can promote wellbeing	Semistructured interviews
Yildizeli et al	Burden, depression and fatigue in caregivers of lung transplantation candidates	Zarit Burden Scale, Beck Depression Inventory, SF-36

Abbreviation: QOL, quality of life.

Caregiver assessment tools have been extrapolated from non–lung transplant populations and have not been validated in caregivers of lung transplant recipients. Capturing the extent of burden in this population remains difficult as caregivers of patients undergoing lung transplantation face unique challenges related to financial burden, fear of transplant complications, relocation strain, and job security, which are not directly incorporated into current assessment questionnaires. Until a caregiver burden assessment tool is validated in the lung transplant population, we recommend using a combination of multiple assessment tools to comprehensively gauge caregiver burden. For example, the Caregiver Reaction Assessment tool and the Zarit-Caregiver Burden Scale assessment can be used to assess multidimensional emotional strain and the COPE index can be used to assess financial strain and social functioning.^50^ We recommend against using generic instruments to screen for anxiety and depression as they are unlikely to accurately portray the multiple facets of caregiver burden in this population.

### Caregiver effects on lung transplant patient outcomes

Our search identified 3 studies evaluating the effects of caregivers on patient outcomes after lung transplantation.^6^, ^51^, ^52^ A retrospective cohort study of 111 lung transplant recipients and their caregivers assessed quality of recipient-caregiver relationships with a primary outcome measure of patients’ perception of self-care agency. Results showed that the quality of the caregiver relationship was a significant predictor for patients’ perception of self-care agency and psychological distress. For every 1-point decrease in the quality of the relationship, self-care agency scores lowered by 12%.^51^ Importantly, perceptions of self-care agency were evaluated rather than actual self-care behavior which may limit interpretation of the true effect on outcomes.

A prospective study in caregivers of cardiothoracic transplant recipients evaluated predictors of patient mortality. QOL was evaluated in caregivers at 2, 7, and 12 months post-transplant and patients were followed for an additional 7 years. Lower QOL scores at 12 months in caregivers were independently associated with recipient mortality. For every 5-point decrease in the general health subscale in caregivers, mortality increased by 10% respectively in patients.^6^

Another retrospective cohort study with 452 patients and their respective caregivers assessing the association between type of caregiver with survival and graft rejection demonstrated that patients who were cared for by a spouse had a significantly greater 1-, 3-, and 5-year survival as compared to those cared for by an adult child. Patients cared for by spouses also survived longer than those cared for by siblings.^52^ This difference may be due to nonspouse caregivers having competing responsibilities such as taking care of their own spouses and children. Adult children may also have major life events, such as marriage, career demands, and raising children, which may reduce the time spent caregiving.^52^

### Interventions to mitigate caregiver strain

Three studies evaluated interventions in caregivers of patients before and after lung transplantation.^22^, ^28^, ^48^ A study of 30 caregivers evaluating mindful meditation in reducing caregiver strain demonstrated that the group that was taught meditation techniques had significantly reduced anxiety after 4 weeks when compared to baseline.^22^ Mindful meditation has also been studied in the non–lung transplant caregiver population with similar improvement in depression and anxiety.^53^

A randomized-controlled trial evaluating the effect of telephone-based psychological services demonstrated significantly higher QOL scores, mood symptoms, and intimacy scores in caregivers of patients who received psychological services compared to the control group.^48^ Lastly, health care hospitality houses, where patients and families live together during prolonged hospitalizations, have improved quality of life in multiple patient populations.^54^, ^55^ A study evaluating the effects of a hospitality house on 1,247 solid-organ transplant recipients/caregivers demonstrated a positive effect on well-being.^28^

Other interventions studied in the non–lung transplant caregiver population include video-conference coaching sessions and web-based interventions.^56^, ^57^ Future studies on caregivers of lung transplant patients are needed to evaluate the effect of web-based interventions, palliative care, and shared caregiving on caregiver and patient outcomes.

### Social determinants of health and caregiver burden

Racial and ethnic minorities, women, and patients in lower socioeconomic status groups are less likely to be referred, evaluated, and waitlisted for organ transplantation.^58^ Living in rural areas and low health literacy are independent risk factors for low likelihood of listing.^59^, ^60^ In addition to decreased rates of referral and listing, lower socioeconomic status poses challenges in finding a caregiver as families may not be able to take time off work for caregiver responsibilities. For those without adequate social support, paid caregivers may be an option though this can be cost-prohibitive and most lung transplant programs will not allow this option. In a review of 51 studies, the annual cost of caregiving in the nontransplant population was estimated to be $30,562.^61^

Informal, unpaid caregivers face significant financial burden due to reduced earnings. The economic cost of informal caregiving in the United States is estimated to be $44 billion.^62^ A study exploring the needs of caregivers in lung transplantation revealed that all caregivers had to stop working during the transplant process to fulfill caregiving duties.^24^ While the Family Medical Leave Act in the United States allows caregivers to take time off, only 12 unpaid weeks are approved in the majority of the states. Strict eligibility criteria must also be met, including providing a 30-day notice, which may be challenging given the unpredictability of post-transplant complications.^63^ Thirteen states provide paid leave programs and offer state-paid support for an average of 8 weeks. However, these programs do not apply to self-employed caregivers or caregivers with cash-only income.^64^

In many European countries, caregiver leave is compensated. In Ireland, leave can be renewed for up to 104 weeks and in the United Kingdom, caregivers can enroll in flexible working hours.^65^ However, financial compensation during leave and part-time work is lower compared to full-time employment.

Lower socioeconomic status is associated with negative patient outcomes, though it is unknown whether access to adequate caregiving is a mediator. A retrospective study of 27,763 adults who underwent lung transplantation between 2005 and 2020 demonstrated that patients who had high levels of socioeconomic distress had increased 5-year mortality.^66^ Access to caregiver support was not independently assessed. Additional studies evaluating the intersection of social determinants of health, access to caregivers, caregiver burden, and patient outcomes are needed in the lung transplant population.

## Discussion

Lung transplant candidates and recipients require significant caregiver support. Though studies comparing caregivers of lung transplant patients to other types of caregivers are not available, many aspects of lung transplant may uniquely burden caregivers. Lung transplantation has the shortest survival rate among solid organ transplantation, and though patients may do well for several years, caregivers will likely need to resume this role after complications arise. Caregivers may also need to change their lifestyle to minimize infection risk and other post-transplant complications. Lastly, refusal of caregiving may deem a potential candidate ineligible which may add significant stress and moral imperative for caregivers.

During the pretransplantation period, caregivers face a higher incidence of mood disorders compared to population norms along with financial burden. During the post-transplantation period, caregivers reported low daily mood and increased physical symptoms. Studies thus far have used multiple assessment tools to evaluate mood disorders, physical symptoms, and financial burden in caregivers. Standardized assessments of caregiver burden, which may require a combination of tools and an assessment of validity in the lung transplant population, should be implemented in future studies.

Few interventions have shown improved outcomes in caregivers of lung transplant patients. Mindful meditation techniques and telephone-based psychological interventions have both been effective in improving symptom burden, though randomized-controlled trials evaluating existing and novel interventions are required. Relief of financial burden with extension of leave, flexible working hours, and paid time off needs to be explored further. The impact of social workers on relieving caregiver strain must also be studied as social workers typically have prolonged contact with caregivers before and after transplant and are aware of caregiver stress related to relationship changes, role overload, and financial strain. Additionally, studies assessing caregiver burden at various time points in the post-transplant period should be conducted as caregiver burden changes throughout the transplant life cycle.

## Conclusions

Lung transplant centers are dependent on the willingness of caregivers to provide care, and without caregivers, eligibility for transplant may be jeopardized. Multiple studies have demonstrated psychological, physical, and financial burdens in the caregiver population. Caregiver-patient relationships and caregiver QOL have been independently associated with patient outcomes. Additional studies are needed in caregivers of lung transplant patients to identify interventions to mitigate caregiver strain and improve caregiver and patient outcomes. Transplant programs must also consider advocating for government-run programs to establish systemic support for paid caregiver leave which will decrease caregiver burden.

## Author contributions

**Keerthana Sankar:** Conceptualization, Data curation, Visualization, Writing – original draft, Writing – reviewing and editing. **Anne Johnson:** Writing – reviewing and editing. **Kathryn McRae:** Writing – reviewing and editing. **Reinaldo Rampolla:** Supervision, Writing – reviewing and editing. **Nicholas A. Kolaitis:** Conceptualization, Supervision, Writing – reviewing and editing.

## Disclosure statement

The authors declare that they have no known competing financial interests or personal relationships that could have appeared to influence the work reported in this paper.

Acknowledgments: None.
